# The Effect of Cryopreserved Human Placental Tissues on Biofilm Formation of Wound-Associated Pathogens

**DOI:** 10.3390/jfb9010003

**Published:** 2018-01-08

**Authors:** Yong Mao, Anya Singh-Varma, Tyler Hoffman, Sandeep Dhall, Alla Danilkovitch, Joachim Kohn

**Affiliations:** 1 New Jersey Center for Biomaterials, Rutgers University, 145 Bevier Rd., Piscataway, NJ 08854, USA; maoy@dls.rutgers.edu (Y.M.); as2220@scarletmail.rutgers.edu (A.S.-V.); 2 Osiris Therapeutics, Inc., Columbia, MD 21046, USA; THoffman@osiris.com (T.H.); SDhall@osiris.com (S.D.); ADanilkovitch@osiris.com (A.D.)

**Keywords:** biofilm, chronic wound, cryopreserved amniotic membrane, cryopreserved umbilical cord tissue, antibacterial

## Abstract

Biofilm, a community of bacteria, is tolerant to antimicrobial agents and ubiquitous in chronic wounds. In a chronic DFU (Diabetic Foot Ulcers) clinical trial, the use of a human cryopreserved viable amniotic membrane (CVAM) resulted in a high rate of wound closure and reduction of wound-related infections. Our previous study demonstrated that CVAM possesses intrinsic antimicrobial activity against a spectrum of wound-associated bacteria under planktonic culture conditions. In this study, we evaluated the effect of CVAM and cryopreserved viable umbilical tissue (CVUT) on biofilm formation of *S. aureus* and *P. aeruginosa*, the two most prominent pathogens associated with chronic wounds. Firstly, we showed that, like CVAM, CVUT released antibacterial activity against multiple bacterial pathogens and the devitalization of CVUT reduced its antibacterial activity. The biofilm formation was then measured using a high throughput method and an ex vivo porcine dermal tissue model. We demonstrate that the formation of biofilm was significantly reduced in the presence of CVAM- or CVUT-derived conditioned media compared to control assay medium. The formation of *P. aeruginosa* biofilm on CVAM-conditioned medium saturated porcine dermal tissues was reduced 97% compared with the biofilm formation on the control medium saturated dermal tissues. The formation of *S. auerus* biofilm on CVUT-conditioned medium saturated dermal tissues was reduced 72% compared with the biofilm formation on the control tissues. This study is the first to show that human cryopreserved viable placental tissues release factors that inhibit biofilm formation. Our results provide an explanation for the in vivo observation of their ability to support wound healing.

## 1. Introduction

Bacterial colonization of surfaces, especially in the form of a biofilm, is a main contributor to delayed wound healing [1,2]. Biofilm represents a community of attached bacteria (often multi-species) protected by a self-secreted extracellular polymeric substance [3]. Biofilms have significant resistance to both antimicrobial agents [4] and host immune defenses [5]. Despite decades of biofilm research, no effective anti-biofilm treatments or point-of-care diagnostic tools to help identify wound biofilm in situ exist [2,6]. In current clinical practices, the physical removal of biofilm by sharp debridement continues to be the most common wound treatment [2,6,7]. After aggressive debridement, the re-formation of biofilms can occur within 24 h and often mature within 72 h [8]. Therefore, prevention of biofilm formation in the wound is critical [6]. Ideally, biofilm prevention and/or disruption therapies should be applied within a narrow window following wound debridement. A panel of experts in clinical and laboratory biofilm research recommend that advanced wound therapies, such as biological wound dressings and growth factors, be applied after debridement and treatment with biofilm disrupting agents [2]. In agreement with these recommendations, an advanced wound therapy with intrinsic ability to prevent or reduce biofilm formation would have significant therapeutic benefits.

Our previous studies demonstrated that CVAM has antibacterial activity against a broad spectrum of pathogens such as ESKAPE (*Enterococcus faecium*, *Staphylococcus aureus*, *Klebsiella pneumoniae*, *Acinetobacter baumannii*, *Pseudomonas aeruginosa* or *Enterobacter aerogenes*) [9]. This data indicates that CVAM retains intrinsic antimicrobial properties of fresh amnion that likely contribute to the positive outcomes observed in clinical studies [10].

Very recently, it has been reported that total protein extracts prepared from fresh amniotic and chorionic membranes inhibited biofilm formation of *Streptococcus pneumonia* [11]. Total human placental extract has also been shown to reduce biofilm formation of *S. aureus* and *P. aeruginosa* [12]. However, the ability of fresh viable placental tissues to produce and release factors that inhibit biofilm formation has not been explored. In the present study, we investigated the effects of CVAM and CVUT on biofilm formation of chronic wound-associated pathogens. As *Staphylococcus aureus* and *Pseudomonas aeruginosa* are the most prominent bacteria present in chronic wounds [13], *S. aureus* and *P. aeruginosa* were used as model bacteria in these studies to monitor biofilm formation on synthetic surfaces or in ex vivo porcine dermal tissues [14,15].

While data exists on CVAM antimicrobial activity, comparable data on CVUT was unavailable. As a first step in the present study, we confirmed that CVUT also possesses antibacterial activity. While CVUT reduced the growth of planktonic *S. aureus*, *P. aeruginosa* and *MRSA* (methicillin resistant *S. aureus*) by more than 5 logs compared with the control, it showed no activity against *E. faecium*, *K. pneumoniae* or *A. baumannii*. Since CVAM and CVUT have been used to treat chronic wounds, the prevalence of biofilm in the chronic wounds prompted us to explore the effect of CVAM or CVUT on the biofilm formation. In addition to a commonly used biofilm formation (on a synthetic surface) assay, we also tested the biofilm formation on an ex vivo porcine dermal tissue, which mimics an in vivo tissue environment. We demonstrate that both CVAM- and CVUT-derived conditioned media can inhibit biofilm formation of *S. aureus* and *P. aeruginosa* on either synthetic surfaces or in porcine dermal tissues, providing support for both as beneficial biomaterials for the management of chronic wounds.

## 2. Results and Discussion

### 2.1. Cryopreserved Placental Tissues Retain the Native Structure and Cell Viability of Fresh Tissues

**Figure 1:**
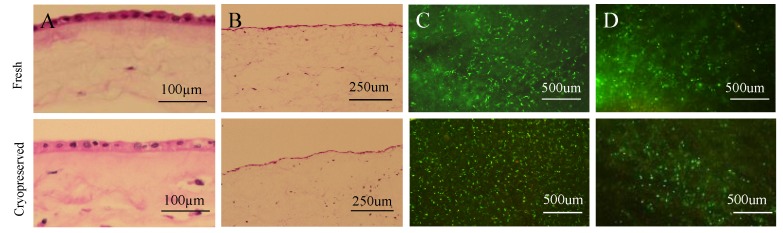
Structure and cell viability of fresh and cryopreserved placental tissues. Fresh amniotic membrane and cryopreserved viable amniotic membrane (**A**) and fresh and cryopreserved umbilical cord tissues (**B**) were analyzed by Haemotoxylin &Eosin staining. Live/Dead staining of cells in both fresh and cryopreserved amniotic membrane (**C**) and fresh and cryopreserved umbilical cord (**D**).

CVAM and CVUT were processed using Osiris Therapeutics’ proprietary cryopreservation methods [9,16]. As shown in Figure 1, both native tissue structure and cell viability were preserved in both cryopreserved tissues.

### 2.2. Antibacterial Activity of CVUT against ESKAPE

The antibacterial activity of CVAM has been demonstrated previously [9]. In the present study, the antibacterial activity of CVUT was evaluated against ESKAPE pathogens as described in Materials and Methods. One-way analysis of variance (ANOVA) was performed between the treatment group (CVAM or CVUT) and assay medium control for each individual bacterial pathogen. A reduction in *S. aureus*, *P. aeruginosa* and *E. aerogenes* growth, expressed as the log difference compared with the growth in the assay medium control (Table 1), was observed in the presence of CVUT conditioned medium. Conversely, no significant difference was detected for *E. faecium*, *K. pneumonia* or *A. baumannii*.

### 2.3. Viable Cells in CVUT Contribute to Antibacterial Activity

**Figure 2:**
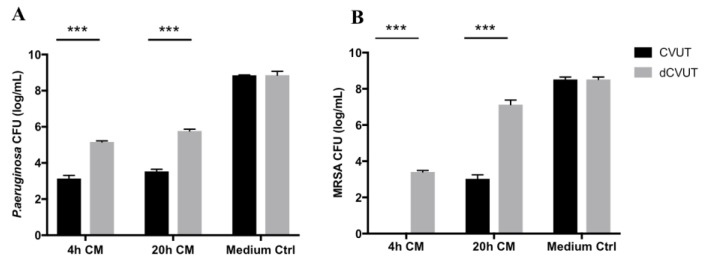
Viable cells contribute to antibacterial activity against *P. aeruginosa* and *MRSA*. Sequentially collected CVUT and dCVUT conditioned media (4 h and 20 h) were prepared as described in Materials and Methods, and effects of conditioned media were tested on the growth of *P. aeruginosa* (**A**) or *MRSA* (**B**). Data are presented as mean ± standard deviation (SD) of CFU (log)/mL. *** *p* < 0.001.

It has been shown that viable cells in CVAM contribute to antibacterial activity: devitalization of CVAM leads to a reduction in antibacterial activity [9,17]. To investigate the contribution of viable cells to CVUT antibacterial activity, CVUT tissue was devitalized by several repeated freeze-thaw cycles. CVUT is composed of umbilical amnion and a proteoglycan-rich matrix, known as Wharton’s jelly, with sparsely distributed endogenous mesenchymal origin cells [18]. To determine the contribution of antibacterial factors present in the matrix versus those released directly from cells, CVUT or devitalized CVUT (dCVUT) conditioned medium was generated by sequential incubation of the tissue in assay medium as described in Materials and Methods. CVUT and dCVUT conditioned media was collected and tested against *P. aeruginosa* (Figure 2A) and *MRSA* (Figure 2B). We found that devitalization of CVUT significantly reduced antibacterial activity against both *P. aeruginosa* and *MRSA*.

Antibacterial activity was detected in conditioned medium collected after 4 h incubation (4 h CM). The significant difference between conditioned media from CVUT and dCVUT suggests that even within the first 4 h, antibacterial factors are released from viable cells in CVUT. However, the observed anti-*P. aeruginosa* or anti-*MRSA* activity of 4 h dCVUT conditioned media suggested that antibacterial factors are also present within umbilical matrix. The activity against *MRSA* in 20 h dCVUT-derived conditioned medium was lower as compared with the activity of 4 h conditioned medium, indicating a depletion of antibacterial factors from the matrix over time. The reduced activity of dCVUT 20 h conditioned medium compared to CVUT 20 h conditioned medium implied that viable cells contribute to sustained release of anti-*S. aureus* factors from CVUT.

### 2.4. Antimicrobial Peptides in CVUT Conditioned Medium

**Figure 3:**
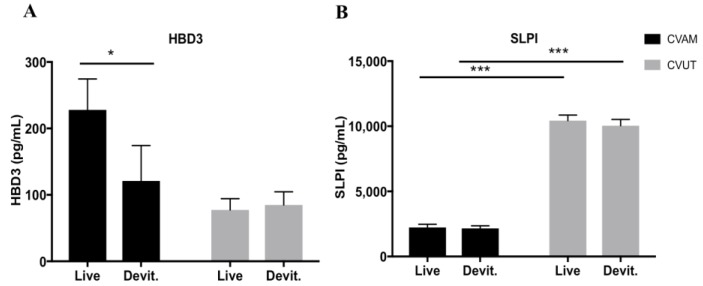
Antimicrobial peptides (AMPs) are present in CVUT and CVAM conditioned media. The levels of human beta-defensin 3 (HBD3) (**A**) and secretory leukocyte protease inhibitor (SLPI) (**B**) in conditioned media were quantified using ELISA. Data are presented as mean ± SD (n = 6). * *p* < 0.05 and *** *p* < 0.001.

Previously, we found that multiple antimicrobial peptides (AMPs), in part, mediate the antibacterial activity of CVAM [17]. In this study, human beta defensins (HBD2 and HBD3) and secretory leukocyte protease inhibitor (SLPI) present in CVUT conditioned medium were quantified by enzyme-linked immunosorbent assay (ELISA) and compared to levels of those in CVAM (Figure 3). Conditioned media were collected from both viable and devitalized forms of CVAM and CVUT after 24 h incubation in assay medium. The levels of HBD2 in CVUT conditioned medium were low and close to the assay detection limit (data not shown). However, HBD3 was detected in both CVUT- and CVAM-derived conditioned media (Figure 3A), although the levels were higher for CVAM as compared to CVUT. Conditioned medium from devitalized CVAM showed reduced levels of HBD3, while no difference in HBD3 was found between conditioned medium derived from viable or devitalized CVUT. In contrast, SLPI levels were significantly higher in CVUT conditioned medium (10.04 ± 0.47 ng/mL) versus CVAM conditioned medium (2.23 ± 0.25 ng/mL) (Figure 3B). Interestingly, devitalization of CVUT had no significant effect on SLPI levels, suggesting that it originates from tissue matrices.

### 2.5. Effect of CVAM on S. aureus and P. aeruginosa Biofilm Formation

**Figure 4:**
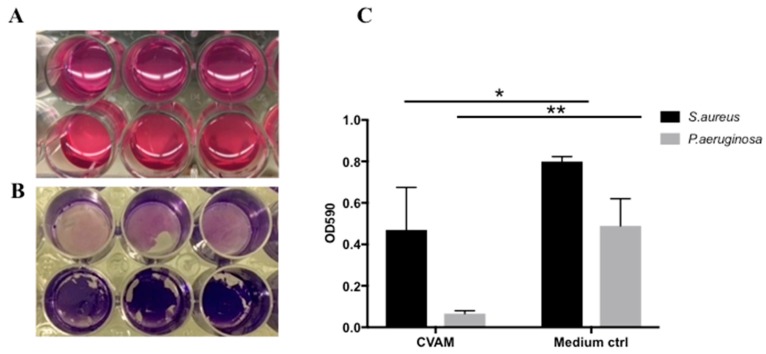
CVAM-derived conditioned medium reduced biofilm formation on synthetic surfaces. (**A**,**B**) Conditioned medium (top row) or assay medium (bottom row) was inoculated with *P. aeruginosa* and incubated for a total of 72 h as described in Materials and Methods. Biofilms present in each well were washed and stained with crystal violet (**B**). (**C**) *S. aureus* and *P. aeruginosa* biofilm formation was quantified by measuring the OD_590_ of the extracted dye solution. Data are presented as mean ± SD (n = 3) of one representative experiment. * *p* < 0.05; ** *p* < 0.01.

Biofilm formation on synthetic surfaces was examined using a modified high-throughput biofilm assay [14]. CVAM-derived conditioned medium or assay medium (control) was inoculated with *S. aureus* or *P. aeruginosa* bacteria in polystyrene culture plates. After 48 h, the biofilms were then stained with crystal violet (Figure 4A,B) and quantified by dissolving crystal violet in 95% ethanol and reading the absorbance at 590 nm. (Figure 4C).

As shown in Figure 4C, biofilm formation of both *S. aureus* and *P. aeruginosa* in conditioned media was reduced as compared to the assay medium control. This reduction was more pronounced with *P. aeruginosa* than with *S. aureus*, consistent with the observation that CVAM has a stronger activity against gram-negative bacteria, such as *P. aeruginosa* [9].

### 2.6. Effect of CVUT on S. aureus and P. aeruginosa Biofilm Formation

**Figure 5:**
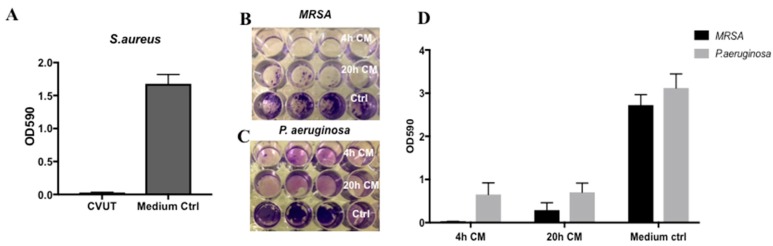
CVUT-derived conditioned media reduced biofilm formation on synthetic surfaces. *MRSA* (**A**) *or P. aeruginosa* (**B**) was used to inoculate conditioned media (4 h CM—top row, 20 h CM—middle row and assay medium—bottom row) and incubated for a total of 72 h. Biofilms were washed and stained with crystal violet. The amount of stain in biofilms of *S. aureus* (**C**) or *MRSA* and *P. aeruginosa* (**D**) was quantified by measuring the absorbance at 590 nm. Data are presented as mean ± SD of one representative experiment (n = 4).

Next, biofilm formation on synthetic surfaces in the presence of CVUT-derived conditioned medium was analyzed using a modified high-throughput biofilm assay [14]. *S. aureus* biofilm formation was evaluated at 72 h and compared with that for assay medium (Figure 5A). *S. aureus* biofilm formation in CVUT-derived conditioned medium was significantly inhibited. In addition, formation of *methicillin-resistant S. aureus* (*MRSA)* biofilm was also evaluated in this study. *MRSA* (Figure 5B) or *P. aeruginosa* (Figure 5C) was used to inoculate CVUT-derived conditioned medium prepared sequentially (4 h CM and 20 h CM) or assay medium (control). Biofilm formation was evaluated at 72 h (Figure 5D).

Biofilm formation of *MRSA* or *P. aeruginosa* was significantly reduced in the presence of both 4 h and 20 h CVUT-derived conditioned media. This inhibitory effect was more pronounced for *MRSA* biofilm formation (Figure 5D), consistent with the selective antibacterial activity of CVUT against *MRSA* (Table 1). Conditioned medium from the 4 h time point had a stronger activity against *MRSA* biofilm formation than that from the 20 h time point (Figure 5D). This observation suggests that additional anti-*MRSA* biofilm factors may be released during the initial 4 h incubation compared with the 20 h incubation.

### 2.7. Effect of CVAM on Biofilm Formation in Porcine Dermal Tissue

**Figure 6:**
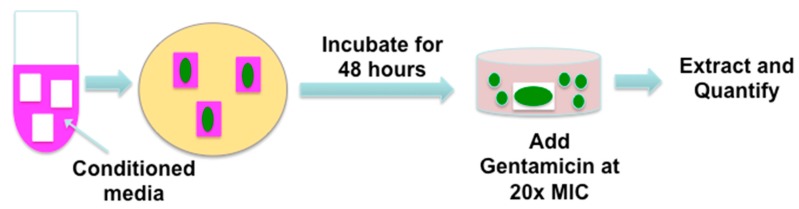
Schematic representation of the porcine dermal tissue explant model. Porcine dermal tissue pieces were saturated in placental-derived conditioned media and added to a Tryptic Soy broth agar (TSA) plate containing gentamicin. Wells containing porcine tissue pieces were inoculated with bacteria and incubated for 48 h, followed by antibiotic treatment to eliminate planktonic bacteria. The bacteria in biofilms were extracted and quantified using a CFU assay.

To investigate biofilm formation in wounds, we utilized an ex vivo biofilm model established by Dr. Schultz’s laboratory [15]. Schematic representation of this porcine dermal tissue explant model is shown in Figure 6.

**Figure 7:**
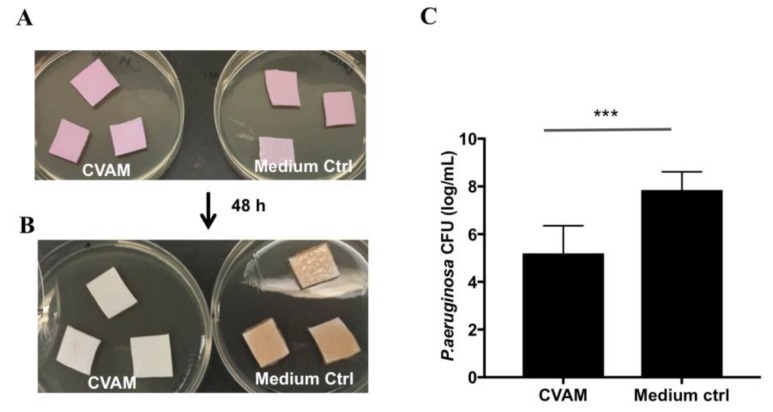
CVAM-derived conditioned media from reduced biofilm formation of *P. aeruginosa* in a porcine dermal tissue model. Porcine dermal tissue pieces treated with conditioned media derived from CVAM or with assay medium (control) (**A**). *P. aeruginosa* was used to inoculate the tissue pieces and incubated for 48 h (**B**). Biofilm bacteria were extracted and quantified by determining the CFUs (**C**). Data are presented as mean ± SD of one representative experiment (n = 3). *** *p* < 0.001.

Porcine dermal tissue pieces were saturated in conditioned media derived from CVAM or assay medium control and added to a tryptic soy agar (TSA) plate containing a 0.5× minimal inhibitory concentration (MIC) of gentamicin (Figure 7A). *P. aeruginosa* was used to inoculate the tissue and incubated for 48 h (Figure 7B). Biofilm bacteria were then extracted and quantified by counting the colony forming units (CFU) (Figure 7C). The CFU of bacteria recovered from CVAM conditioned medium-treated tissues was 2.7 logs lower than the CFU of bacteria recovered from control tissues. Therefore, *P. aeruginosa* biofilm formation was significantly reduced (97%) in the presence of the CVAM-derived conditioned medium. Conversely, no significant inhibition of *S. aureus* or *MRSA* biofilm formation was observed in this model in the presence of CVAM-derived conditioned medium (data not shown).

### 2.8. Effect of CVUT on Biofilm Formation in the Porcine Dermal Tissue Explant Model

**Figure 8:**
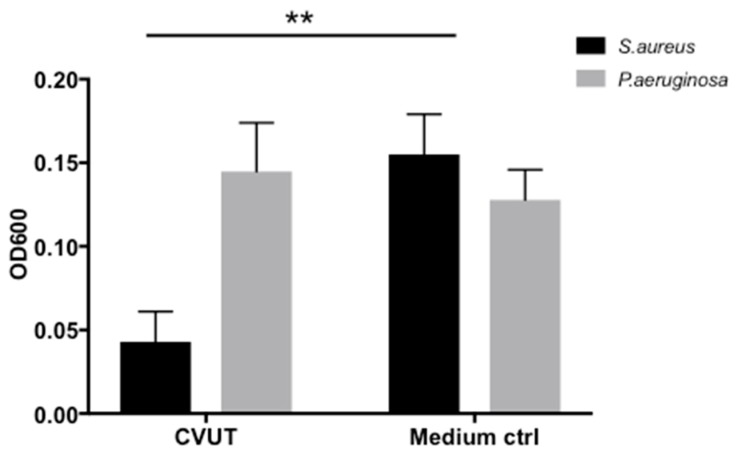
CVUT-conditioned media reduced biofilm formation on porcine dermal tissues. Porcine dermal tissue pieces saturated with conditioned media derived from CVUT or with assay medium (control) were added to a TSA agar plate containing gentamicin. *S. aureus* or *P. aeruginosa* was used to inoculate the tissue pieces. Resulting biofilms were extracted and bacteria were quantified by measuring the OD_600_. Data are presented as mean ± SD of one representative experiment (n = 4).

Next, we evaluated the effect of CVUT on biofilm formation in the porcine dermal tissue model (Figure 8). CVUT-conditioned medium was collected after 24 h. Dermal tissue pieces were treated with conditioned media or assay medium (control), followed by inoculation with *S. aureus* or *P. aeruginosa* bacteria and incubated at 37 °C for 48 h. Following removal of planktonic bacteria, the bacteria in biofilms were extracted. Since the extracts looked turbid, instead of quantifying the CFU of each extract, the turbidity at OD_600_ of each extract was measured (Figure 8). As shown in Figure 8, formation of *S. aureus* biofilm was significantly reduced in this model. However, no activity against *P. aeruginosa* biofilm formation was observed. While there was a delay in *P. aeruginosa* growth at the 24 h time point (data not shown), the additional 48 h incubation period minimized this effect. It is possible that this dermal tissue, which mimics necrotic or non-viable tissue, serves as a protein source to support the growth of bacteria, leading to biofilm formation, if antibacterial activity is not sufficient to control initial bacterial growth.

## 3. Materials and Methods

### 3.1. Preparation of CVAM and CVUT Samples and Conditioned Medium

Human term placental tissues were obtained from commercial tissue agencies and processed at Osiris Therapeutics, Inc. (Columbia, MD, USA) following the proprietary manufacturing procedure [17]. Devitalized CVUT samples were prepared by five cycles of freeze (liquid nitrogen) and thaw (37 °C). All samples were stored at −80 °C prior to experiments. On the day of each experiment, tissues were thawed in a room temperature water bath for 3–5 min. Tissues were removed from a storage container (cryobag or cryovial), placed in a sterile basin, and washed with sterile PBS. Preparation of conditioned medium from CVAM or CVUT was carried out by incubation in a sterile 50 mL conical tube containing assay medium (1 mL per 4 cm^2^ of CVAM and 1 mL per 1 cm^2^ of CVUT) consisting of Dulbecco’s Modified Eagle Medium (DMEM; Invitrogen, Calsbad, CA, USA) and 10% fetal bovine serum (FBS; Atlanta Biologicals, Flowery Branch, GA, USA), followed by shaking at 37 °C at 5% CO_2_ and 95% humidity. Conditioned media was collected at 24 h for single time point experiments. For the preparation of sequential conditioning medium, CVUT was incubated in assay medium (1 mL per 1 cm^2^) for 4 h. The medium was collected and labeled as “4 h CM”. The same volume of fresh assay medium was added to CVUT tissue and incubated for 20 h to generate “20 h CM”. All conditioned media collected was either used immediately or stored at −80 °C prior to testing.

### 3.2. Bacterial Cultures and Preparation of Inoculums

Clinical isolates of bacterial strains *Enterococcus faecium* ATCC^®^ 51559™, *Staphylococcus aureus* ATCC^®^ 25923™, *Klebsiella pneumoniae* ATCC^®^ 700603, *Acinetobacter baumannii* ATCC^®^ 49466™, *Pseudomonas aeruginosa* ATCC^®^ 15692™, *Enterobacter aerogenes* ATCC^®^ 49469™, *methicillin-resistant Staphylococcus aureus* (*S. aureus*) (MRSA; ATCC^®^ BAA-1720) and *Vancomycin-resistant Enterococci* (VRE; ATCC 700221) were purchased from American Type Culture Collection (ATCC), (Gaithersburg, MD, USA) and maintained following instructions provided by the supplier. Preparation of bacterial inoculum was performed as described previously [9]. Briefly, bacteria were cultured in tryptic soy broth at 37 °C with shaking until the absorbance optical densities measured in the range of 0.2 to 0.6 at a wavelength of 600 nm. The number of colony forming units (CFU) for each strain was estimated based on an OD_600_ = 1.0, which corresponds to 10^9^ CFU/mL. To prepare the inoculum, the bacterial stock solution was diluted with either assay medium or conditioned medium to approximately 100 CFU of bacteria/mL. For each experiment, the actual CFU of each inoculum was determined by preparing serial dilutions and plating onto tryptic soy broth agar plates.

### 3.3. Liquid Antibacterial Activity Assay

100 CFU of bacteria were cultured with 1 mL of assay medium or conditioned medium and incubated at 37 °C with shaking for 24 h. Serial dilutions were prepared of each culture and plated onto tryptic soy broth agar plates. CFUs were counted after overnight plate incubation at 37 °C.

### 3.4. Detection of Human Beta-Defensins (HBDs) and Secretory Leukocyte Protease Inhibitor (SLPI) Using ELISA

CVUT or devitalized CVUT were cultured in assay medium for 24 h. Conditioned media was collected after 24 h incubation and analyzed for human beta-defensin 2 (HBD2), human beta-defensin 3 (HBD3) and human SLPI using ELISA kits (Human BD-2 Mini ABTS ELISA kit and Human BD-3 Mini ABTS ELISA kit, PeproTech, Rocky Hill, NJ, USA) and SLPI ELISA kit (MBS266338, MyBioSource, San Diego, CA, USA) according to the manufacturer’s instructions.

### 3.5. Biofilm Formation on Synthetic Surfaces

The biofilm formation assay was modified from a high throughput biofilm assay [14]. 1 × 10^3^ CFU of *P. aeruginosa* (ATCC 15692) was added to each well of a 24-well tissue culture plate (T1024 Denville Scientific Inc., Denville, NJ, USA) containing 1 mL of either conditioned medium or assay medium (control). Plates were incubated at 37 °C for 48 h without shaking. To kill planktonic bacteria [15], the medium was removed and 0.5 mL of fresh assay medium containing 125 µg/mL gentamicin was added to each well to achieve a final concentration equal to 20 × of MIC of *P. aeruginosa* (6.25 µg/mL), followed by another 24 h incubation period. After incubation, the biomass of biofilm was quantified [19]. Briefly, the media was removed from each well, and each well was washed twice with 1 mL PBS. The plates were air-dried at room temperature for 10 min. Next, 500 µL of 0.1% crystal violet was added to each well and incubated for 30 min. The dye was removed and each well was washed extensively with dH_2_O to remove any free dye. After air-drying, each well was treated with 1 mL of 95% ethanol to dissolve the remaining biofilms. The resulting biofilm-ethanol solution was transferred to a 96-well plate (with dilutions when necessary). The absorbance at OD_590_ of a 100 µL aliquot of each sample was measured using a microtiter plate reader spectrophotometer (Spark 10M, TECAN, Männedorf, Switzerland).

### 3.6. Porcine Dermal Tissue Procurement

Fresh porcine dermal tissue was harvested at Farm to Pharm, LLC. (Warren, NJ, USA). A Yorkshire breed pig (~130 lb, female) was sacrificed according to the USDA approved procedure. Hair was shaved using an electronic clipper and shaved areas were quickly sprayed with 70% ethanol and dried using Kimwipes. Dermatome (Integra, Plainsboro, NJ, USA) was used to remove the epidermis. Dermal tissue strips were harvested by hand-held blades, soaked in bottles containing 0.2% peracetic acid (PAA) and transported back to the laboratory. In the biosafety cabinet, the fat was removed from the dermis. The tissue was cut into 1.5 cm × 1.5 cm pieces, treated with 0.5% PAA for 3 h and washed extensively with sterile water for 24 h (with at least five changes of water). The tissue was used immediately or stored in PBS at 4 °C up to seven days prior to each experiment.

### 3.7. Biofilm Formation in Ex Vivo Porcine Dermal Tissues

The biofilm formation assay was modified from an ex vivo biofilm model established by Dr. Schultz’s group [15]. Sterile porcine dermal tissue pieces were soaked in conditioned media derived from CVAM or CVUT (4 mL of medium for three pieces of tissue) or in assay medium (4 mL of medium for three pieces of tissue) at room temperature for 4 h with gentle shaking. After this coating step, the tissue was added to tryptic soy agar plates (TSA) containing 3.125 µg/mL of gentamicin. A 10 µL aliquot of bacteria (1 × 10^5^ CFU/mL) was carefully spread onto the surface of each piece of porcine dermal tissue. The plates were incubated at 37 °C for 48 h, and three pieces of tissue were transferred to 5 mL of PBS containing 125 µg/mL gentamicin (6-well plate) and incubated for another 24 h followed by three washes with PBS. To extract bacteria, three pieces of tissue were transferred to a 50 mL conical tube using 10 mL of PBS. Tissues samples were vortexed at maximum speed for 10 min (1 min with 1 min of rest). Quantitative assessment of biofilms was performed using turbidity (OD_600_) or CFU of extracted bacteria.

### 3.8. Cell Viability Testing

The LIVE/DEAD^®^ Viability/Cytotoxicity Kit (ThermoFisher Cat# L3224, Bridgewater, NJ, USA) was used to analyze cell viability of CVAM and CVUT according to the procedure described previously [9]. Briefly, tissue samples were thawed and rinsed twice with PBS and stained with 4 µM calcein AM (green) and 2 µM ethidium homodimer-1 (red) according to the manufacturer’s protocol. Stained CVAM samples were then analyzed using the EVOS FL Auto 2 (Thermofisher AMAFD2000, Bridgewater, NJ, USA) directly on a glass slide and stained CVUT samples were sectioned to thin slices and analyzed using the EVOS FL Auto 2.

### 3.9. Histological Analyses

CVAM, CVUT, fresh AM and fresh UT were fixed in 4% paraformaldehyde. Tissue embedding, sectioning and staining were performed by Histoserv Inc. (Germantown, MD, USA) using standard protocols for hematoxylin and eosin (H&E).

### 3.10. Statistical Analysis

Each independent experiment contained three or more biological repeat samples (n ≥ 3). Descriptive statistics included mean and SD. One-way ANOVA with a Tukey’s multiple comparisons test was performed to determine statistical significance between experimental groups (Prism Software, Version 7, GraphPad Software, Inc., La Jolla, CA, USA). Differences were considered significant at a *p* value of <0.05.

## 4. Conclusions

In this study, we investigated the antibacterial activity of CVUT against ESKAPE pathogens. We demonstrate that CVUT possesses antibacterial activity that is mediated by soluble factors released into the culture medium. CVUT antimicrobial activity is likely mediated by soluble factors present within the extracellular matrix, available for immediate release, and by soluble factors secreted by viable cells over time. CVUT is selectively active against *S. aureus*, *MRSA*, *P. aeruginosa* and *E. aerogenes* and has no activity against *E. faecium*, *K. pneumonia* or *A. baumannii*. CVAM also has antibacterial activity against selected types of bacteria, yet the antimicrobial activity profile is different from that of CVUT. This observation suggests that amnion and umbilical tissue may contain and secrete a different spectrum of antimicrobial factors. Consistent with this observation, we found that levels of human beta defensins and SLPI in CVAM- and CVUT-derived conditioned media are significantly different. We also found that CVAM- and CVUT-derived conditioned media reduced biofilm formation of *P. aeruginosa* and *S. aureus* on a polystyrene surface. Using an ex vivo porcine dermal tissue model, we demonstrated a reduction in the formation of *P. aeruginosa* biofilm in the presence of CVAM conditioned medium and a reduction in the formation of *S. aureus* biofilm in the presence of CVUT conditioned medium. Our finding is consistent with the positive clinical outcomes of using CVAM or CVUT in treating chronic wounds.

## Figures and Tables

**Table 1:** Antibacterial activities of CVAM and CVUT against ESKAPE pathogens. The growth reduction of each bacterial pathogen was calculated as log_10_CFU (assay medium control)—log_10_CFU (CVAM or CVUT). ^TM^: TradeMark and CFU: colony forming units * *p* < 0.01.

ESKAPE	ATCC#	Gram Stain	Growth Reduction in CVAM (log)	Growth Reduction in CVUT (log)
***E**nterococcus faecium *	51559™	Positive	0.6 ± 0.3 *	0.1 ± 0.1
***S**taphylococcus aureus *	25923™	Positive	2.6 ± 1.5	7.5 ± 0.5
***K**lebsiella pneumoniae *	700603™	Negative	5.1 ± 1.7	0.7 ± 0.1
***A**cinetobacter baumannii *	49466™	Negative	5.1 ± 0.9	0.7 ± 0.4
***P**seudomonas aeruginosa *	15692™	Negative	6.6 ± 1.8	5.0 ± 1.2
***E**nterobacter aerogenes *	49469™	Negative	3.6 ± 1.4	3.3 ± 0.6
***VRE***	700221™	Positive	0.6 ± 0.1 *	0.1 ± 0.2
***MRSA***	BAA-1720™	Positive	5.8 ± 0.9	6.6 ± 0.3
